# Low Probability of Initiating *nirS* Transcription Explains Observed Gas Kinetics and Growth of Bacteria Switching from Aerobic Respiration to Denitrification

**DOI:** 10.1371/journal.pcbi.1003933

**Published:** 2014-11-06

**Authors:** Junaid Hassan, Linda L. Bergaust, I. David Wheat, Lars R. Bakken

**Affiliations:** 1 Department of Environmental Sciences, Norwegian University of Life Sciences, Ås, Norway; 2 Department of Geography, University of Bergen, Bergen, Norway; Boston University, United States of America

## Abstract

In response to impending anoxic conditions, denitrifying bacteria sustain respiratory metabolism by producing enzymes for reducing nitrogen oxyanions/-oxides (NO_x_) to N_2_ (denitrification). Since denitrifying bacteria are non-fermentative, the initial production of denitrification proteome depends on energy from aerobic respiration. Thus, if a cell fails to synthesise a minimum of denitrification proteome before O_2_ is completely exhausted, it will be unable to produce it later due to energy-limitation. Such entrapment in anoxia is recently claimed to be a major phenomenon in batch cultures of the model organism *Paracoccus denitrificans* on the basis of measured e^−^-flow rates to O_2_ and NO_x_. Here we constructed a dynamic model and explicitly simulated actual kinetics of recruitment of the cells to denitrification to directly and more accurately estimate the recruited fraction (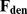). Transcription of *nirS* is pivotal for denitrification, for it triggers a cascade of events leading to the synthesis of a full-fledged denitrification proteome. The model is based on the hypothesis that *nirS* has a low probability (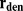, h^−1^) of initial transcription, but once initiated, the transcription is greatly enhanced through positive feedback by NO, resulting in the recruitment of the transcribing cell to denitrification. We assume that the recruitment is initiated as [O_2_] falls below a critical threshold and terminates (assuming energy-limitation) as [O_2_] exhausts. With 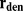 = 0.005 h^−1^, the model robustly simulates observed denitrification kinetics for a range of culture conditions. The resulting 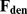 (fraction of the cells recruited to denitrification) falls within 0.038–0.161. In contrast, if the recruitment of the entire population is assumed, the simulated denitrification kinetics deviate grossly from those observed. The phenomenon can be understood as a ‘bet-hedging strategy’: switching to denitrification is a gain if anoxic spell lasts long but is a waste of energy if anoxia turns out to be a ‘false alarm’.

## Author Summary

In response to oxygen-limiting conditions, denitrifying bacteria produce a set of enzymes to convert / to N_2_ via NO and N_2_O. The process (denitrification) helps generate energy for survival and growth during anoxia. Denitrification is imperative for the nitrogen cycle and has far-reaching consequences including contribution to global warming and destruction of stratospheric ozone. Recent experiments provide circumstantial evidence for a previously unknown phenomenon in the model denitrifying bacterium *Paracoccus denitrificans:* as O_2_ depletes, only a marginal fraction of its population appears to switch to denitrification. We hypothesise that the low success rate is due to ***a)*** low probability for the cells to initiate the transcription of genes (*nirS*) encoding a key denitrification enzyme (NirS), and ***b)*** a limited time-window in which NirS must be produced. Based on this hypothesis, we constructed a dynamic model of denitrification in *Pa. denitrificans*. The simulation results show that, within the limited time available, a probability of 0.005 h^−1^ for each cell to initiate *nirS* transcription (resulting in the recruitment of 3.8–16.1% cells to denitrification) is sufficient to adequately simulate experimental data. The result challenges conventional outlook on the regulation of denitrification in general and that of *Pa. denitrificans* in particular.

## Introduction

A complete denitrification pathway includes the dissimilatory reduction of nitrate (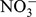) through nitrite (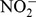), nitric oxide (NO), and nitrous oxide (N_2_O) to di-nitrogen (N_2_). Typically, the genes encoding reductases for these nitrogen oxyanions/-oxides (NO_x_) are not expressed constitutively but only in response to O_2_ depletion, making denitrification a facultative trait [1]. Hence, during anoxic spells, the process enables denitrifying bacteria to sustain respiratory metabolism, replacing O_2_ by NO_x_ as the terminal electron (e^−^) acceptors. Since permanently anoxic environments lack available NO_x_, denitrification is confined to sites where O_2_ concentration fluctuates, such as biofilms, surface layers of sediments, and drained soil (which turns anoxic in response to flooding).

### From modelling denitrifying communities as a homogenous unit to a model of regulation of denitrification in an individual strain

Denitrification is a key process in the global nitrogen cycle and is also a major source of atmospheric N_2_O [2]. A plethora of biogeochemical models have been developed for understanding the ecosystem controls of denitrification and N_2_O emissions [3]. A common feature of these models is that the denitrifying community of the system (primarily soils and sediments) in question is treated as one homogenous unit with certain characteristic responses to O_2_ and 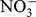 concentrations. This simplification is fully legitimate from a pragmatic point of view, but in reality any denitrifying community is composed of a mixture of organisms with widely different denitrification regulatory phenotypes [4]. Modelling has been used to a limited extent to analyse kinetic data for various phenotypes (See [5] and references therein) and for understanding the accumulation of intermediates [6]. To our knowledge, however, no attempts have been made to model the regulation during transition from aerobic to anaerobic respiration in individual strains, despite considerable progress in the understanding of their regulatory networks. It would be well worth the effort, since the regulatory phenomena at the cellular level provide clues as to how denitrification and NO and N_2_O emissions therefrom are regulated in intact soils [7]. Explicit modelling of the entire denitrification regulatory network, however, would take us beyond available experimental evidence, with numerous parameters for which there are no empirical values. Considering this limitation, here we have constructed a simplified model to investigate if a stochastic transcriptional initiation of key denitrification genes (*nirS*) could possibly explain peculiar kinetics of e^−^-flow as *Paracoccus denitrificans* switch from aerobic to anaerobic respiration [4], [8].

Although denitrification is widespread among bacteria, the α-proteobacterium *Pa. denitrificans* is the ‘paradigm’ model organism in denitrification research. Recent studies [4], [8], [9] have indicated a previously unknown phenomenon in this species that, in response to O_2_ depletion, only a marginal fraction (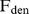) of its entire population appears to successfully switch to denitrification. In these studies, however, 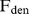 is inferred from rates of consumption and production of gases (O_2_, NO_x_, and N_2_), and a clear hypothesis as to the underlying cause of the low 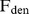 is also lacking. To fill these gaps, we formulated a refined hypothesis addressing the underlying regulatory mechanism of the cell differentiation in response to O_2_ depletion. On its basis, we constructed a dynamic model and explicitly simulated the actual kinetics of recruitment of the cells from aerobic respiration to denitrification. The model adequately matches batch cultivation data for a range of experimental conditions [4], [8] and provides a direct and refined estimation of 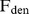. The exercise is important for understanding the physiology of denitrification in general and of *Pa. denitrificans* in particular and carries important implications for correctly interpreting various denitrification experiments.

### Regulation of denitrification in terms of relevance to fitness

Generally, the transcription of genes encoding denitrification enzymes is inactivated in the presence of O_2_. A population undertaking denitrification typically responds to full aeration by completely shutting down denitrification and immediately initiating aerobic respiration [10]. Thus, O_2_ controls denitrification at transcriptional as well as metabolic level, and both have a plausible fitness value. The transcriptional control minimises the energy cost of producing denitrification enzymes, and the metabolic control maximises ATP (per mole electrons transferred) because the mole ATP per mole electrons transferred to the terminal e^−^-acceptor is ∼50% higher for aerobic respiration than for denitrification [10].

Denitrification enzymes produced in response to an anoxic spell are likely to linger within the cells under subsequent oxic conditions (although, this has not been studied in detail), ready to be used if O_2_ should become limiting later on. However, these enzymes will be diluted by aerobic growth, since the transcription of their genes is effectively inactivated by O_2_. Hence, a population growing through many generations under fully oxic conditions will probably be dominated by the cells without intact denitrification proteome. When confronted with O_2_ depletion, such a population will have to start from scratch, i.e., transcribe the relevant genes, translate mRNA into peptide chains (protein synthesis by ribosomes) and secure that these chains are correctly folded by the chaperones, transport the enzymes to their correct locations in the cell, and insert necessary co-factors (e.g., Cu, Fe, or Mo). In *E. coli* grown under optimal conditions, the whole process from the transcriptional activation to a functional enzyme takes ≤20 minutes [11] and costs significant amount of energy (ATP).

Synthesis of denitrification enzymes is rewarding if anoxia lasts long and NO_x_ remains available, but it is a waste of energy if anoxia is brief. Since the organisms cannot sense how long an impending anoxic spell will last, a ‘bet-hedging strategy’ [12] where one fraction of a population synthesises denitrification enzymes while the other does not may increase overall fitness.

### A delayed response to O_2_ depletion may lead to entrapment in anoxia

Most, if not all, denitrifying bacteria are non-fermentative and completely rely on respiration to generate energy [13], [14]. This implies that their metabolic machinery will run out of energy whenever deprived of terminal e^−^-acceptors. When [O_2_] falls below some critical threshold, the cells will ‘sense’ this and start synthesising denitrification proteome, utilising energy from aerobic respiration [10]. However, if O_2_ is suddenly exhausted or removed, the lack of a terminal e^−^-acceptor will create energy limitation, restraining the cells from enzyme synthesis, hence, entrapping them in anoxia. This was clearly demonstrated by Højberg *et al.* [15], who used silicone immobilised cells to transfer them from a completely oxic to a completely anoxic environment. Such a rapid transition is unlikely to occur in nature; however, the experiment illustrates one of the apparent perils in the regulation of denitrification: the cells that respond too late to O_2_ depletion will be entrapped in anoxia, unable to utilise alternative electron acceptors for energy conservation and growth.

**Figure 1:**
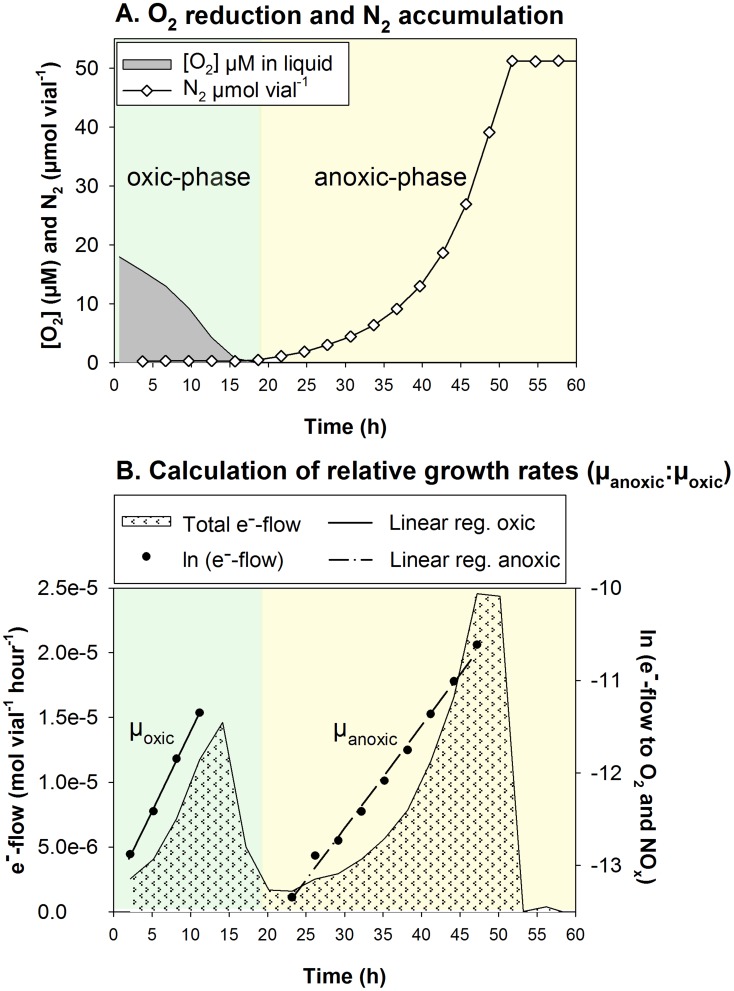
Data generated by batch cultivation of *Pa. denitrificans* [4] (redrawn). As the cells transited from oxic to anoxic conditions (Panel A), Bergaust *et al.* [4] observed a severe depression in the total e^−^-flow rate (i.e., to O_2_+NO_x_, Panel B), which was taken to indicate that only a fraction of the cells switched to anaerobic respiration (denitrification). Had all of the cells switched, the total e^−^-flow would have carried on increasing without such a depression. The depression was followed by an exponential increase in the e^−^-flow rate, which was ascribed to anaerobic growth of a small fraction (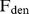) of the cells that escaped entrapment in anoxia and carried on growing by denitrification.

Højberg *et al.*'s [15] observations have largely been ignored in the research on the regulation of denitrification, and it is implicitly assumed that, in response to O_2_ depletion, all cells in cultures of denitrifying bacteria will switch to denitrification. Contrary to this, however, Bergaust *et al.* [4], [8], [16] followed by Nadeem *et al.* [9] proposed that in batch cultures of *Pa. denitrificans*, only a small fraction of all cells is able to switch to denitrification. During transition from oxic to anoxic conditions, they observed a severe depression in the total e^−^-flow rate (i.e., to O_2_+NO_x_, see Fig. 1), which was estimated on the basis of measured gas kinetics. Had all of the cells switched to denitrification as O_2_ exhausted, the total e^−^-flow rate would have carried on increasing, without such a depression. The depression was followed by an exponential increase in the e^−^-flow rate, which was tentatively ascribed to anaerobic growth of a small 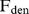 (fraction recruited to denitrification). It was postulated that this fraction escaped entrapment in anoxia by synthesising initial denitrification proteins within the time-window when O_2_ was still present, whereas the majority of the cells (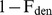) failed to do so, thus remained unable to utilise NO_x_.

### The core hypothesis: A low probability of initiating *nirS* transcription seems to drive the cell differentiation

#### Autocatalytic transcription of denitrification genes

**Figure 2:**
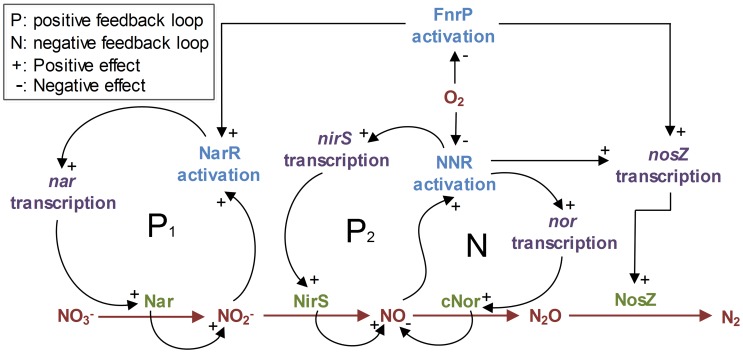
The regulatory network of denitrification in *Pa. denitrificans.* In *Pa. denitrificans*, denitrification is driven by four core enzymes: Nar (nitrate reductase encoded by the *nar* genes), NirS (nitrite reductase encoded by *nirS*), cNor (NO reductase encoded by *nor*), and NosZ (N_2_O reductase encoded by *nosZ*). The transcription of these genes is regulated by, at least, three FNR-type proteins, which are sensors for O_2_ (FnrP), 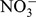/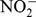 (NarR), and NO (NNR). NarR and NNR facilitate product-induced transcription of the *nar* and *nirS* genes (see positive-feedback loops), where NNR also counteracts the NO accumulation (negative-feedback loop) [10], [17], [18]. Circumstantial evidence suggests that O_2_ inactivates NNR (grey dashed link) [20], and NirS is also unlikely to be functional in the presence of high O_2_ concentrations. Hence, for our modelling we hypothesise that the probability of an autocatalytic transcriptional activation of *nirS* is zero until O_2_ falls below a critical concentration 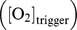. When O_2_ falls below 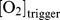, the initial *nirS* transcription is possibly mediated through a minute pool of intact NNR, crosstalk with other factors, or through non-biological traces of NO found in an 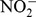-supplemented medium. Regardless of the exact mechanism(s), once *nirS* transcription is initiated, it will be substantially enhanced by spikes of internal NO emitted from the first molecules of NirS (the positive-feedback loop). The activated positive-feedback will also induce *nor* and *nosZ* transcription via NNR (although, the latter can also be induced independently by FnrP [19]), facilitating the synthesis of a full-fledged denitrification proteome. Our model assumes that such recruitment to denitrification will occur with a low probability. We further assume that the recruitment will only be possible as long as a minimum of O_2_ 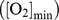 is available because the production of the first molecules of NirS will depend on energy from aerobic respiration.

In *Pa. denitrificans*, denitrification is driven by four core enzymes: Nar (membrane-bound nitrate reductase), NirS (cytochrome *cd_1_* nitrite reductase), cNor (nitric oxide reductase), and NosZ (nitrous oxide reductase, see Fig. 2). The transcriptional regulation of genes encoding these enzymes (*nar*, *nirS*, *nor* and *nosZ*, respectively) involves, at least, three FNR-type proteins acting as sensors for O_2_ (FnrP), 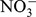/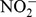 (NarR), and NO (NNR) [10], [17], [18]. NarR and NNR facilitate product-induced transcription of the *nar* and *nirS* genes: When anoxia is imminent, the low [O_2_] is sensed by FnrP, which in interplay with NarR induces *nar* transcription. NarR is activated by 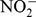 (and/or probably by 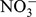); thus once a cell starts producing traces of 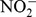, *nar* expression becomes autocatalytic. The transcription of *nirS* is induced by NNR, which requires NO for activation; thus once traces of NO are produced, the expression of *nirS* also becomes autocatalytic. In contrast, the transcription of *nor* is substrate (NO) induced via NNR, while *nosZ* is equally but independently induced by NNR and FnrP [19]. Here we are concerned with the dynamics that start with the transcription of *nirS*, since the experimental treatments that we simulated were not supplemented with 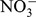 but various concentrations of 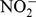 only (Table 1).

**Table 1:** The simulated experiment of Bergaust *et al* [4], [8].

Batch No.	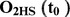 (vol. %)*	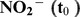 (mM)
1	∼0	0.2
2	∼0	1
3	∼0	2
4	1	0.2
5	1	1
6	1	2
7	7	0.2
8	7	1
9	7	2

*Targeted values for initial O_2_ in the headspace (where the headspace vol. = 70 mL). The actual initial O_2_ measured in the 0, 1, and 7% treatments was 0.012–0.19, 1.2–1.66, 6.6–6.8 vol.%, respectively. The O_2_ present in the ∼0% treatments was due to traces of O_2_ left behind despite various cycles of evacuation of the headspace air and subsequent flushing of the vials with helium (He-washing).

#### Low probability of initiating *nirS* transcription

The transcription of *nirS* is known to be suppressed by O_2_ [4], [8], but the exact mechanism remains unclear. Circumstantial evidence suggests that it is due to O_2_ inactivating NNR [20] (dashed link in Fig. 2), but this is not necessary to explain the repression of NirS. There are several mechanisms through which high O_2_ concentrations may restrain NirS activity, i.e., through post-transcriptional regulation, direct interaction with the enzyme, or due to competition for electrons. Regardless of the exact mechanism(s), the ultimate consequence is the elimination of the positive feedback via NO and NNR. When O_2_ falls below a critical threshold, facilitating NirS activity, this positive feedback would allow the product of a single transcript of *nirS* to induce a subsequent burst of *nirS* transcription in response to NO. Such ‘switches’ in gene expression by positive-feedback loops are not uncommon in prokaryotes, and they have been found to result in cell differentiation because the initial transcription is stochastic with a relatively low probability [21].

Our model assumes such stochastic recruitment to denitrification, triggered by an initial *nirS* transcription occurring with a low probability. This initial transcription is possibly mediated by a minute pool of intact NNR and/or through crosstalk with other factors, such as FnrP. A 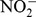-supplemented medium contains non-biologically formed traces of NO which, once diffused into the cells while O_2_ is low, will activate background levels of NNR and, thereby, may also increase the probability of triggering *nirS* transcription.

For this modelling exercise, we do not need a full clarification of the mechanisms involved but only to assume that the probability of an autocatalytic transcriptional activation of *nirS* would be practically zero as long as O_2_ concentration is above a certain threshold. This assumption is backed by empirical data indicating that NO is not produced to detectable levels before O_2_ concentration falls below a critical threshold [8], [22]. For O_2_ concentrations below this threshold, the model assumes a low (but unknown) probability for each cell to initiate the autocatalytic transcription of *nirS*, paving the way for the rest of the denitrification proteome.

#### O_2_ is required for the initial production of NirS

We further assume that the recruitment to denitrification will only be possible as long as a minimum of O_2_ is available because the synthesis of first molecules of NirS will depend on energy from aerobic respiration.

#### Can NO produced within one cell help activate the autocatalytic transcription of *nirS* in the neighbouring cells?

It is perhaps less obvious that the autocatalytic transcriptional activation of *nirS* takes place only within the NO-producing cell because NO diffuses easily across membranes [23]. However, the average distance between the cells in a culture with 10^9^ cells mL^−1^ (roughly the numbers that we are dealing with) is ∼10 µm, which is ∼10 times the diameter of a cell. This implies that an NO molecule produced by a cell has a much higher probability to react with and activate the NNR inside the same cell than to do so in another one.

### Modelling the cell differentiation

To represent the batch cultivation conducted by Bergaust *et al.* [4], [8], the model explicitly simulates growth of two sub-populations, one *with* denitrification enzymes (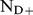) and the other *without* (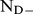); both equally consume O_2_, but 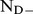 cannot reduce NO_x_ to N_2_. Once oxygen concentration in the liquid 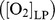 falls below a critical level 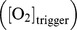 [22], the cells within 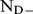 are assumed to initiate *nirS* transcription (and thereby ensure recruitment to 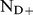) with a rate described by a probabilistic function: 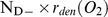 (cells h^−1^), where 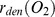 is assumed to be an 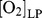 dependent probability (h^−1^) for any cell within 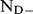 to initiate *nirS* transcription (leading to a full denitrification capacity). When 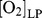 falls below 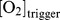, 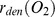 triggers and holds a constant value as long as 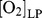 is above a critical minimum 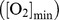. For 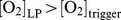, 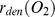 is zero (assuming the inactivation of NNR by O_2_); 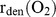 is also zero for 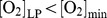 (assuming the lack of energy for protein synthesis).

The recruitment of 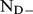 to 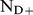 is simulated as an instantaneous event; thus, the model does not take into account the time-lag between the initiation of *nirS* transcription and the time when the transcribing cell has become a fully functional denitrifier. This simplification is based on the evidence that this lag is rather short. Experiments with *E. coli* [11] under optimal conditions suggest lags of ∼20 minutes between the onset of transcription and the emergence of a functional enzyme. In *Pa. denitrificans* [8], [22], the lag observed between the emergence of denitrification gene transcripts and the subsequent gas products suggests that the time required for synthesising the enzymes is within the same range.

### Employing the model to understand ‘diauxic lags’ between the aerobic and anaerobic growth-phases

In a series of experiments with denitrifying bacteria (*Pseudomonas denitrificans*, *Pseudomonas fluorescens*, *Alcaligenes eutrophus* and *Paracoccus pantotrophus*) [24]–[26], oxic cultures were sparged with N_2_ to remove O_2_ and were monitored by measuring optical density (OD_550_). All the strains except *Ps. fluorescens* went through a conspicuous *‘diauxic lag: a period of little or no growth’* [26]; the OD remained practically constant during the lag period, lasting 4–30 hours, which was eventually followed by anaerobic growth.

To understand the diauxic lag, Liu *et al.* [24] used the common assumption that *all* cells would eventually switch to denitrification. They constructed a simulation model based on the assumption that all the cells contained a minimum of denitrification proteome (even after many generations under oxic conditions). This minimum would allow them to produce more denitrification enzymes when deprived of O_2_, albeit very slowly due to energy limitation. The time taken to effectively produce adequate amounts of denitrification enzymes ( = the diauxic lag) was taken to be a function of the initial amounts of these enzymes per cell. Although their model may possibly explain short time-lags, it appears unrealistic for lag phases as long as 10–30 hours [25] because to produce such long lags, conceivably, the initial enzyme concentration would be less than one enzyme molecule per cell, which is mathematically possible but biologically meaningless.

The model presented in this paper provides an alternative explanation for the apparent diauxic lags: a sudden shift from fully oxic to near anoxic conditions (by sparging with N_2_) would leave the medium with only traces of O_2_, which would be quickly depleted due to aerobic respiration. As a consequence, the available time for initiating the synthesis of denitrification proteome would be marginal, allowing only a tiny fraction (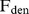) of the cells to switch to denitrification. This marginal fraction would grow exponentially from the very onset of anoxic conditions, but it would remain practically undetectable as measured (OD) for a long time, creating the apparent 4–30 h lag. The length of the lag depends on the fraction of the cells switching to denitrification. To demonstrate this alternative explanation, we adjusted our model to the reported conditions and simulated the experiment of Liu *et al* [24]. The model produced qualitatively similar ‘diauxic lags’ in the simulated cell density (OD), although the time length of the lag could be anything (depending on assumptions regarding the residual O_2_ after sparging, which was not measured).

## Materials and Methods

### An overview of the modelled experiment: Batch incubations in gas-tight vials

**Figure 3:**
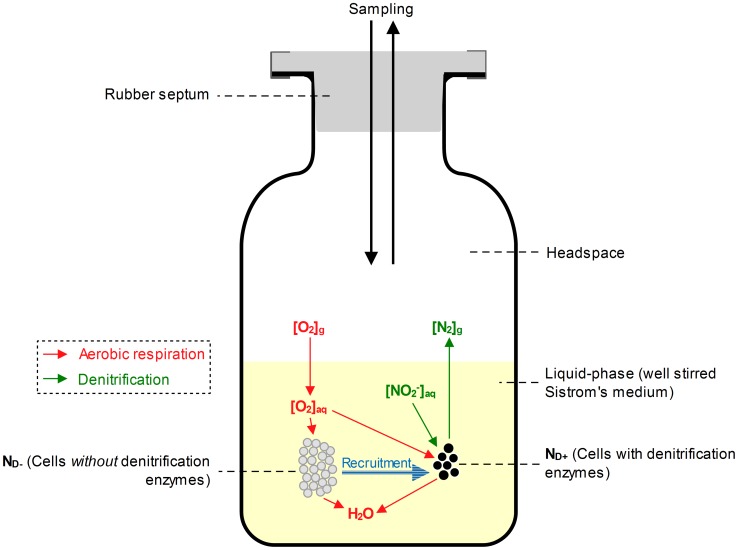
An overview of the modelled system: batch incubation in a gas-tight vial. The experiment: The stirred Sistrom's medium [27] was inoculated with aerobically grown *Pa. denitrificans* cells, which were provided with different concentrations of O_2_ and 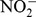 (g or aq with a chemical species-name represents gaseous or aqueous, respectively). O_2_ is consumed by respiration, driving its transport from the headspace to the liquid. Once the aerobic respiration becomes limited, the cells may switch to denitrification (recruitment), reducing 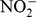 to N_2_ via the intermediates NO and N_2_O (not shown). For monitoring O_2_, CO_2_, N_2_, NO and N_2_O, a robotised incubation system [28] was used, which automatically takes samples from the headspace by piercing the rubber septum. Each sampling removes a fraction (3–3.4%) of all gases in the headspace, but it also involves a marginal leakage of O_2_ and N_2_ into the vial (as indicated by the two-way arrows at the top of the figure). The model: The model operates with two sub-populations: one without and the other with denitrification enzymes (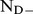 and 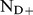, respectively). Both consume O_2_ if present, but 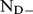 cannot reduce NO_x_. The 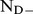 cells may be recruited to the 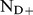 pool as 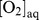 falls below a critical threshold. The rate of recruitment (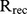) is modelled as a probabilistic function: 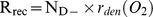 (cells h^−1^), where 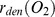 represents an O_2_ dependent specific-probability (h^−1^) for any 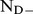 cell to initiate *nirS* transcription (leading to the synthesis of a full-fledged denitrification proteome).

Bergaust *et al.* [4], [8] studied aerobic and anaerobic respiration rates in *Paracoccus denitrificans* (DSM413). The cells were incubated (at 20°C) as stirred batches in 120 mL gastight vials, containing 50 mL Sistrom's medium [27] (Fig. 3). The medium was supplemented with various concentrations of KNO_3_ or KNO_2_. Prior to inoculation, air in the headspace was replaced with He to remove O_2_ and N_2_ (He-washing), followed by the injection of no, 1, or 7 headspace-vol.% O_2_. Finally, each vial was inoculated with ∼3×10^8^ aerobically grown cells.

#### Treatments selected for simulation

Only 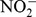-supplemented treatments (Table 1) were selected for this modelling exercise for two reasons. First, 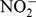 was not monitored; hence, results of the 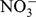-supplemented treatments could not provide exact estimates of anaerobic respiration rates (due to an unknown transient accumulation of 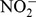). Second, by excluding the treatments requiring Nar, we could single out and focus on the regulation of the other key enzyme NirS.

#### Aerobic respiration followed by denitrification

O_2_ diffused from the headspace to the liquid (Fig. 3), where the cells consumed it before switching to denitrification: the stepwise reduction of 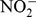 to N_2_ via the intermediates NO and N_2_O (not shown). Headspace concentrations of gases were monitored by frequent sampling (every 3 hours). A typical result is shown in Fig. 1A, illustrating the increasing rate of O_2_ consumption until depletion, followed by transition to denitrification. The denitrification rate increased exponentially till all the 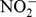 present in the medium was recovered as N_2_. The medium contained ample amounts of carbon substrate (34 mM succinate) to support the consumption of all available electron acceptors.

#### Sampling procedure

To monitor O_2_, CO_2_, NO, N_2_O, and N_2_ in the headspace for respiring cultures, Bergaust *et al.* [4], [8] used a robotised incubation system, which automatically takes samples from the headspace by piercing the rubber septum (Fig. 3). The auto-sampler is connected to a gas chromatograph (GC) and an NO analyser (For details, see [28]). The system uses peristaltic pumping, which removes a fraction (3–3.4%) of all the gases in the headspace and then reverses the pumping to inject an equal amount of He into the headspace, thus maintaining ∼1 atmosphere pressure inside the vial. Sampling also involves a marginal leakage of O_2_ and N_2_ into the headspace (∼22 and ∼60 nmol per sampling, respectively) through tubing and membranes of the injection system.

#### Calculation of gases in the liquid

Concentrations of gases in the liquid were calculated using solubility of each gas at the given temperature (20°C), assuming equilibrium between the headspace and the liquid. However, the O_2_ consumption rate was so high that to calculate [O_2_] in the liquid, its transport rate (from the headspace to the liquid) had to be taken into account.

### An overview of the model

The model effectively represents the physical phenomena mentioned above, so as to ensure that the simulation results match the measured data for the right reasons. Net effect of sampling (dilution and leakage) is included in the simulation of O_2_ kinetics at the reported sampling times. Transport of O_2_ between the headspace and the liquid is modelled using an empirically determined transport coefficient and the solubility of O_2_ in water at 20°C. To simulate the metabolic activity (O_2_ consumption and N_2_ production) and growth, the model divides the cells into two sub-populations: one without and the other with denitrification enzymes (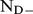 and 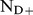 pools, respectively, see Fig. 3). Both equally consume O_2_ if present, but 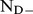 cannot reduce 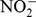 to N_2_. Those 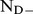 cells that, in response to O_2_ depletion, are able to initiate *nirS* transcription (see Fig. 2) are recruited to the 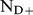 pool, where 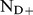 = 0 prior to the recruitment. The recruitment rate (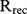) is modelled according to a probabilistic function described below (Eqs. 7–8).

The model ignores sampling effect on N_2_ (leakage and loss), thus calculating the cumulative N_2_ production as if no sampling took place. That is because the experimentally determined N_2_ accumulation (which is to be compared with the model predictions) was already corrected for the net sampling effect.

**Figure 4:**
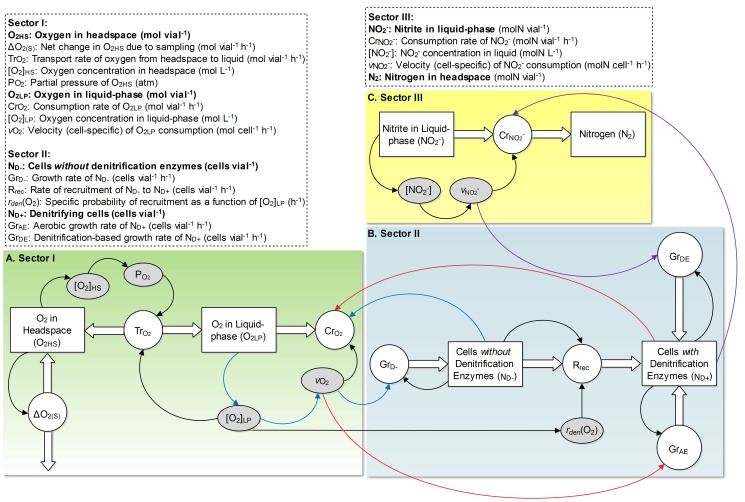
A stock and flow diagram of the model's structure. The squares represent the state variables, the circles the rate of change in the state variables, the shaded ovals the auxiliary variables, the arrows dependencies between the variables, and the edges represent flows into or out of the state variables. **A.** The panel represents the structure that governs the O_2_ kinetics. Briefly, it shows that O_2_ in the vial's headspace (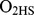) is transported (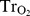) to the liquid-phase (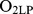), where it is consumed (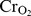) by both 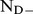 and 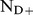 populations with an identical cell-specific velocity of O_2_ consumption (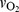). 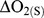 represents net marginal changes in 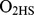 due to sampling. **B.** The panel represents the structural basis for population dynamics of the cells without (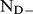) and with (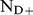) denitrification enzymes. Briefly, it shows that both the populations are able to grow by aerobic respiration (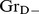 and 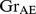, respectively). The growth rate of 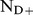, however, is primarily based on denitrification (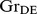). Initially, 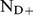 = 0 and is populated through recruitment (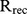) of the cells from 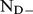, where the recruitment is a function of 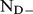 and an [O_2_] dependent specific-probability of the recruitment 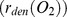 for any 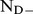 cell. **C.** The panel represents the structural basis for the 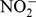/N_2_ kinetics. Briefly, it illustrates that 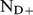 control the consumption rate of 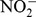 (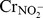), recovered as N_2_, in proportion to a cell-specific velocity of 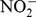 consumption (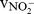).

The model is developed in Vensim DSS 6.2 Double Precision (Ventana Systems, Inc. http://vensim.com/) using techniques from the field of system dynamics [29]. The model is divided into three sectors: I. O_2_ kinetics, II. Population dynamics of 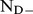 and 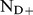, and III. Denitrification kinetics (Fig. 4).

### Sector I: O_2_ kinetics

Structural-basis for the O_2_ kinetics is mapped in Fig. 4A: the squares represent the state variables, the circles the rate of change in the state variables, the shaded ovals the auxiliary variables, the arrows mutual dependencies between the variables, and the edges represent flows into or out of the state variables. Briefly, Fig. 4A (left to right) shows that O_2_ in the vial's headspace (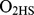) is transported (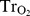) to the liquid-phase (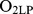), where it is consumed (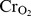) by both the 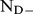 and 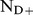 populations (lacking and carrying denitrification enzymes, respectively) in proportion to an identical cell-specific velocity of O_2_ consumption (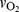). 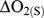 represents net marginal changes in 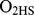 due to sampling. Below we present equations and a detailed explanation of the structural components shown for this sector.

#### O_2_ in the headspace

(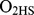, mol vial^−1^) is initialised by measured initial concentrations (Table 1) and modelled as a function of transport (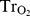) between the headspace and the liquid [28]:
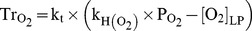
(1)


Units: *mol vial^−1^ h^−1^*

where 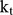 (L vial^−1^ h^−1^) is the empirically determined coefficient for the transport of O_2_ between the headspace and the liquid (See Table 2 for parametric values and their sources), 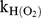 (mol L^−1^ atm^−1^) is the solubility of O_2_ in water at 20°C, 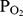 (atm) is the partial pressure of O_2_ in the headspace, and 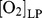 (mol L^−1^) is the O_2_ concentration in the liquid-phase 
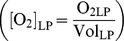
.

**Table 2:** Model parameters.

	Description	Value	Units	Reference
**Sector I: O_2_ Kinetics**				
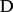	Dilution: the fraction of O_2_ replaced by He during sampling	0.035	Unitless	[28]
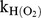	Solubility of O_2_ in water (20°C)	0.00139	mol L^−1^ atm^−1^	[37]
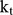	The O_2_ transport coefficient between headspace and liquid	1.62	L vial^−1^ h^−1^	[28]
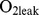	O_2_ leakage into the vial during each sampling	2.04×10^−8^	mol vial^−1^	[28]
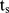	The time taken to complete each sampling	0.017	h	[28]
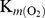	The half saturation constant for O_2_ consumption	2.5×10^−7^	mol L^−1^	Model-based estimation
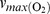	The maximum cell-specific velocity of O_2_ consumption	1.33×10^−15^	mol cell^−1^ h^−1^	[4], [8]
**Sector II: Population dynamics of the cells without (** 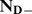 **) and with (** 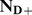 **) denitrification proteome**				
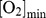	[O_2_] in the liquid below which the recruitment to 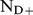 halts	1×10^−9^	mol L^−1^	Assumption
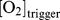	[O_2_] below which the recruitment to 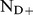 triggers	9.75×10^−6^	mol L^−1^	[22]
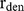	The specific-probability of recruitment of a cell to 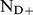	0.0052	h^−1^	Model-based estimation
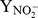	The growth yield per molN 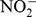	5.79×10^13^	cells molN^−1^	[4], [8]
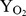	The growth yield per mol O_2_	15×10^13^	cells mol^−1^	[4], [8]
**Sector III: Denitrification Kinetics**				
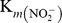	The half saturation constant for 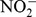 reduction	4×10^−6^	molN L^−1^	[33], [34]
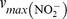	The maximum cell-specific velocity of 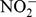 reduction	1.83×10^−15^	molN cell^−1^ h^−1^	[4], [8]
**General**				
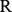	Universal gas constant	0.083	L atm K^−1^ mol^−1^	–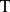	Temperature	293.1	K	[4], [8]
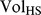	Headspace volume	0.07	L vial^−1^	[4], [8]
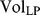	Liquid-phase volume	0.05	L vial^−1^	[4], [8]

In addition, changes in 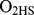 due to sampling are included at the reported sampling times. The robotised incubation system [28] used in the experiment monitors gas concentrations by sampling the headspace, where each sampling alters the concentrations in a predictable manner: a fraction of 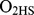 is removed and replaced by He (dilution), but the sampling also results in a marginal leakage of O_2_ through the tubing and membranes of the injection system. Eq. 2 shows how the model calculates the *net* change in 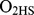 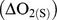 as a result of each sampling:
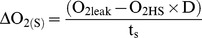
(2)


*mol vial^−1^ h^−1^*

where 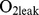 (mol vial^−1^) is the O_2_ leakage into the headspace, 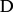 (dilution) is the fraction of 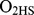 replaced by He, and 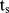 (h) is the time taken to complete each sampling. 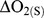 is negative if 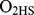 is greater than 0.58 µmol vial^−1^ and marginally positive if it is less than that.

#### O_2_ in the liquid-phase

(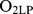, mol vial^−1^, see Fig. 4A) is initialised by assuming equilibrium with 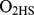 at the time of inoculation 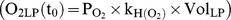. 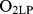 is modelled as a function of its transport into the liquid (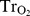, Eq. 1) and consumption rate (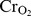, mol vial^−1^ h^−1^), where the latter is modelled as a function of total cell numbers and the cell-specific velocity of O_2_ consumption:
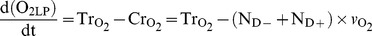
(3)


*mol vial^−1^ h^−1^*

where 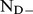 and 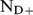 (cells vial^−1^, see Sector II for details) are the cells without and with denitrification enzymes, respectively, and 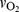 (mol cell^−1^ h^−1^) is the cell-specific velocity of O_2_ consumption. Thus, we assume that the 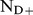 and 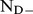 cells have the same potential to consume O_2_.


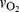
 is modelled as a Michaelis-Menten function of O_2_ concentration:
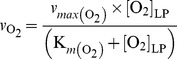
(4)


*mol cell^−1^ h^−1^*

where 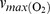 (mol cell^−1^ h^−1^) is the maximum cell-specific velocity of O_2_ consumption (determined under the actual experimental conditions), 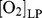 (mol L^−1^) is the O_2_ concentration in the liquid-phase, and 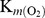 (mol L^−1^) is the half saturation constant for O_2_ reduction.

### Sector II: Population dynamics of the cells without (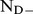) and with (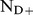) denitrification proteome

Fig. 4B represents the structure governing the population dynamics of 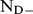 and 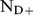. Briefly, the figure shows that both the populations are able to grow by aerobic respiration (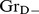 and 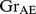, respectively). Initially, 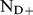 = 0 and is populated through recruitment (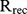) of the cells from the 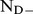 pool, where the recruitment is a product of 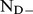 and an [O_2_] dependent specific-probability (h^−1^) of the recruitment (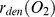, see Eqs. 7–8). The growth rate of 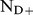 is primarily based on denitrification (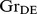), but the 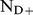 cells that are recruited before O_2_ is completely exhausted also grow by consuming the remaining traces of O_2_. Below we present equations and a detailed explanation of the structural components shown for this sector.

#### The pool of the cells lacking denitrification proteome

The pool of the cells lacking denitrification proteome (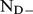) is initialised with 3×10^8^ cells vial^−1^. The population dynamics of 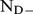 are modelled as:
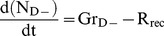
(5)


*cells vial^−1^ h^−1^*

where 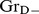 (cells vial^−1^ h^−1^) is the (aerobic) growth rate, and 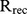 (cells vial^−1^ h^−1^, Eq. 7) is the rate of recruitment of 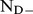 to the 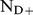 pool.


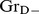
 is modelled as:
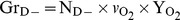
(6)


*cells vial^−1^ h^−1^*

where 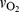 (mol cell^−1^ h^−1^, Eq. 4) is the cell-specific velocity of O_2_ consumption, and 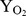 (cells mol^−1^) is the cell yield per mole of O_2_ (determined under the actual experimental conditions).

#### The rate of recruitment

The rate of recruitment (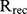, see Fig. 4B) of the cells from 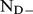 to 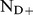 is modelled as:
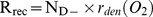
(7)


**Figure 5:**
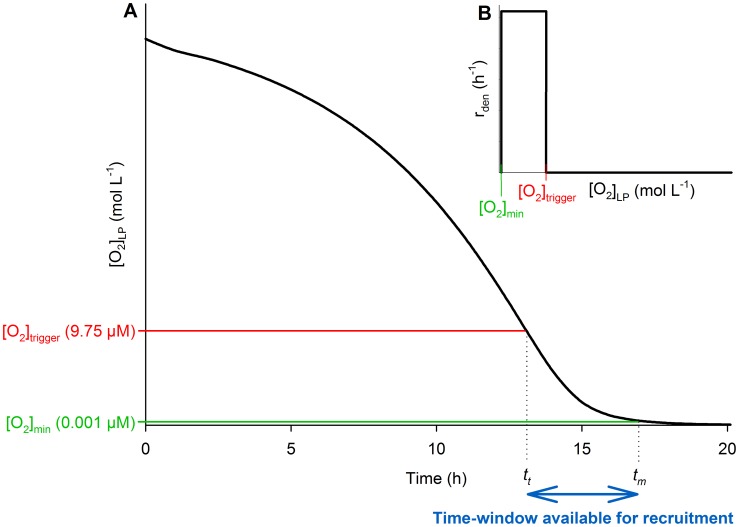
Modelling of 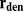
(h^−1^) as a function of 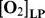. **A.** The panel shows the O_2_ concentration in the liquid-phase 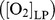 falling as a result of aerobic respiration. **B.** The panel shows the probability for a cell to switch to denitrification (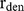, h^−1^) modelled as a function of 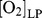. 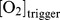 (Panels A & B) is the concentration below which 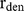 is assumed to trigger (due to withdrawal of the transcriptional control of O_2_ on denitrification [22]), whereas 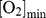 is assumed to be the concentration below which 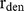 terminates (due to lack of energy for enzyme synthesis). The double-headed arrow (at the bottom of Panel A) illustrates the limited time-window (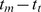) available for the cells to switch to denitrification.

*cells vial^−1^ h^−1^*

where 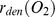 (h^−1^) represents the conditional specific-probability for any 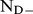 cell to be recruited to denitrification, modelled as a function of O_2_ concentration in the liquid-phase (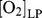, see Fig. 5):
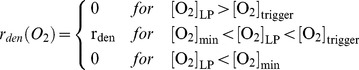
(8)


*h^−1^*

where 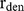 (h^−1^) is a constant representing the specific-probability of the recruitment, 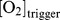 is the O_2_ concentration above which the transcription of *nirS* is effectively suppressed by O_2_, and 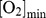 is the O_2_ concentration assumed to provide minimum energy for the initial transcription to result in functional NirS. Once the first molecules of NirS are produced while 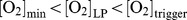, the transcription of *nirS* will be greatly enhanced through positive feedback by NO, paving the way for a full-scale production of denitrification proteome [10] (See Introduction and Fig. 2 for details).


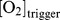
 ( = 9.75×10^−6^ mol L^−1^) is the empirically determined 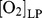 at the outset of NO accumulation: Bergaust *et al.* [8] estimated 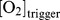 between 0.1–12 µM, but recent *Pa. denitrificans* batch incubation data have provided a more precise estimate between 8.8–10.7 µM (average = 9.75 µM) [22].

As for 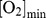, we lack empirical basis for determining the parameter value, but sensitivity of the model to this parameter was tested (See Results/Discussion). Our simulations were run with 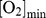 = 1×10^−9^ mol L^−1^, which would sustain an aerobic respiration rate equivalent to 0.4% of the empirically determined 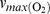 (assuming our estimated 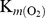 = 2.5×10^−7^ mol L^−1^, Table 2).

As modelled, the time-window for the recruitment to denitrification depends on the time taken to deplete 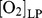 from 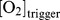 to 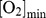 (Fig. 5); for obvious reasons, the length of this time-window depends on the cell density.

The lag observed between the emergence of denitrification gene transcripts and the subsequent gas products is as short as 20 minutes [8], [22], which is insignificant in the sense that the estimations of 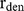 and 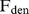 will not be affected by including it in the model. Therefore, the recruitment (Eq. 7) is modelled as an instantaneous event.

#### Calculation of 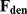: The fraction of the cells recruited to denitrification


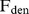
 is calculated based on the integral of the recruitment (Eq. 7):
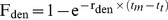
(9)


*Dimensionless*

where 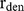 (h^−1^, see Eqs. 7–8 and Fig. 5) is the specific-probability for the recruitment of a cell to denitrification, 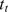 is the time when [O_2_] in the liquid falls below 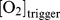 (the concentration below which 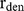 triggers), and 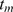 is the time when [O_2_] in the liquid falls below 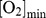 (the concentration below which 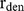 is assumed to be zero). Hence, effectively, 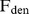 expresses the probability for any cell to switch to denitrification within the time-frame 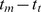.

#### The pool of the cells carrying denitrification proteome

The pool of the cells carrying denitrification proteome (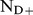, see Fig. 4B) is initialised with zero cells, and its population dynamics are modelled as:
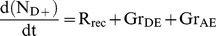
(10)


*cells vial^−1^ h^−1^*

where 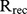 (cells vial^−1^ h^−1^, Eq. 7) is the recruitment rate, 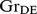 (cells vial^−1^ h^−1^) the denitrification-based growth and 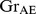 (cells vial^−1^ h^−1^) the aerobic growth rate.


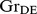
 is modelled as:
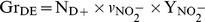
(11)


*cells vial^−1^ h^−1^*

where 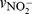 (molN cell^−1^ h^−1^, see Eq. 15) is the cell-specific velocity of 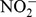 reduction, and 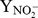 (cells molN^−1^) is the growth yield per molN of 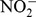 as the e^−^-acceptor (determined under the actual experimental conditions).

The 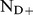 cells are assumed to have the same ability as 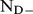 to grow by aerobic respiration; their aerobic growth rate is formulated as:
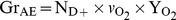
(12)


*cells vial^−1^ h^−1^*

where 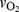 (mol cell^−1^ h^−1^, see Eq. 4) is the cell-specific velocity of O_2_ consumption, and 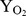 (cells mol^−1^) is the growth yield per mole of O_2_ as the e^−^-acceptor.

### Sector III: Denitrification kinetics

The structure controlling the denitrification kinetics is mapped in Fig. 4C. Briefly, the figure shows that the cells with denitrification proteome (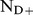) control the consumption rate of 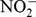 (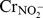), recovered as 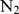, in proportion to a cell-specific velocity of 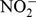 consumption (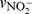). The denitrification intermediates NO and N_2_O are not explicitly modelled, as they accumulated to miniscule concentrations only [4], [8].

#### 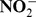 and 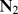


The 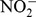 pool (molN vial^−1^) is initialised by measured initial concentrations (Table 1), and the 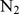 pool is initialised with zero molN vial^−1^. 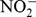 and N_2_ kinetics are modelled as:
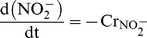
(13)


*molN vial^−1^ h^−1^*

where 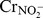 is the consumption rate of 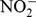:
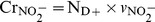
(14)


*molN vial^−1^ h^−1^*

where 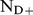 (cells vial^−1^) represents the denitrifying cells, and 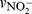 (molN cell^−1^ h^−1^) is the cell-specific velocity of 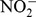 reduction, which is modelled as a function of 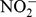 using the Michaelis-Menten equation:
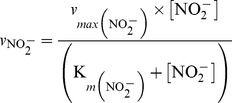
(15)


*molN cell^−1^ h^−1^*

where 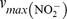 (molN cell^−1^ h^−1^) is the maximum cell-specific velocity of 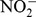 consumption (determined under the actual experimental conditions), 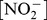 (molN L^−1^) is the 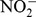 concentration in the liquid-phase, and 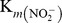 (molN L^−1^) is the half saturation constant for 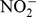 reduction.

See Table 2 for a summary of the parametric values and their sources and Table 3 for the initial values assigned to the state variables.

**Table 3:** Initial values for the state variables.

	Symbol	Value	Units	Reference
**Sector 1: O2 Kinetics**				
Initial O_2_ in the headspace	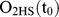	See Table 5	mol vial^−1^	[4], [8]
Initial O_2_ in the liquid-phase	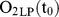	Equilibrium with 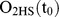	mol vial^−1^	Assumption
**Sector II: Population dynamics of the cells without (** 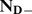 **) and with (** 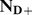 **)denitrification proteome**				
The initial number of cells	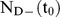	3×10^8^	cells vial^−1^	[4], [8]
The initial number of denitrifying cells	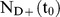	0	cells vial^−1^	Assumption
**Sector III: Denitrification Kinetics**				
Initial 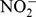 in the liquid-phase	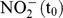	See Table 5	molN vial^−1^	[4], [8]
Initial N_2_ in the headspace	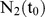	0	molN vial^−1^	[4], [8]

### Parameterisation

Most of the parameter values used in the model are well established in the literature (See Table 2). However, somewhat uncertain parameters include 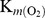, 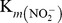, 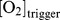, and the assumed parameter 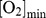:

#### 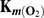


*Pa. denitrificans* has three alternative terminal oxidases [30] with 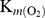 ranging from nM to µM [31], [32], so we decided to estimate 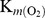 by fitting our model to the data. Unfortunately, Bergaust *et al.'s* [4], [8] ∼0% O_2_ treatments data, for which 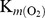 is relevant, has technical problems (needle clogging and/or high O_2_ leakage during sampling). Therefore, we estimated 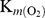 ( = 2.5×10^−7^ mol L^−1^) by aptly simulating our model against another ∼0% O_2_ data-set produced by batch cultivations of *Pa. denitrificans* under similar experimental conditions [22].


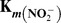
 is given in the literature as 4–5 µM [33], [34]. The model, however, does not show any considerable sensitivity to this parameter even within a range as wide as 0.1–10 µM because the simulated experiments were operating with much higher [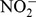].


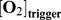
 ( = 9.75×10^−6^ mol L^−1^) is empirically determined as the 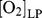 at the outset of NO accumulation: Bergaust *et al.* [8] estimated 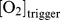 between 0.1–12 µM, but recent batch incubation data from *Pa. denitrificans* have provided a more precise estimate in the range 8.8–10.7 µM (average = 9.75 µM) [22]. The model, however, is not sensitive to 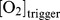 within the latter range because of a high velocity of O_2_ depletion.


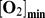
 ( = 1×10^−9^ mol L^−1^) is assigned an arbitrary low value, since we lack any empirical estimation/data to support it. To compensate for the uncertainty, we conducted a sensitivity analysis exploring the consequences of increasing or decreasing 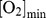 by one order of magnitude (See Results/Discussion).

## Results/Discussion

### The specific-probability (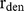, h^−1^) of recruitment of a cell to denitrification

**Figure 6:**
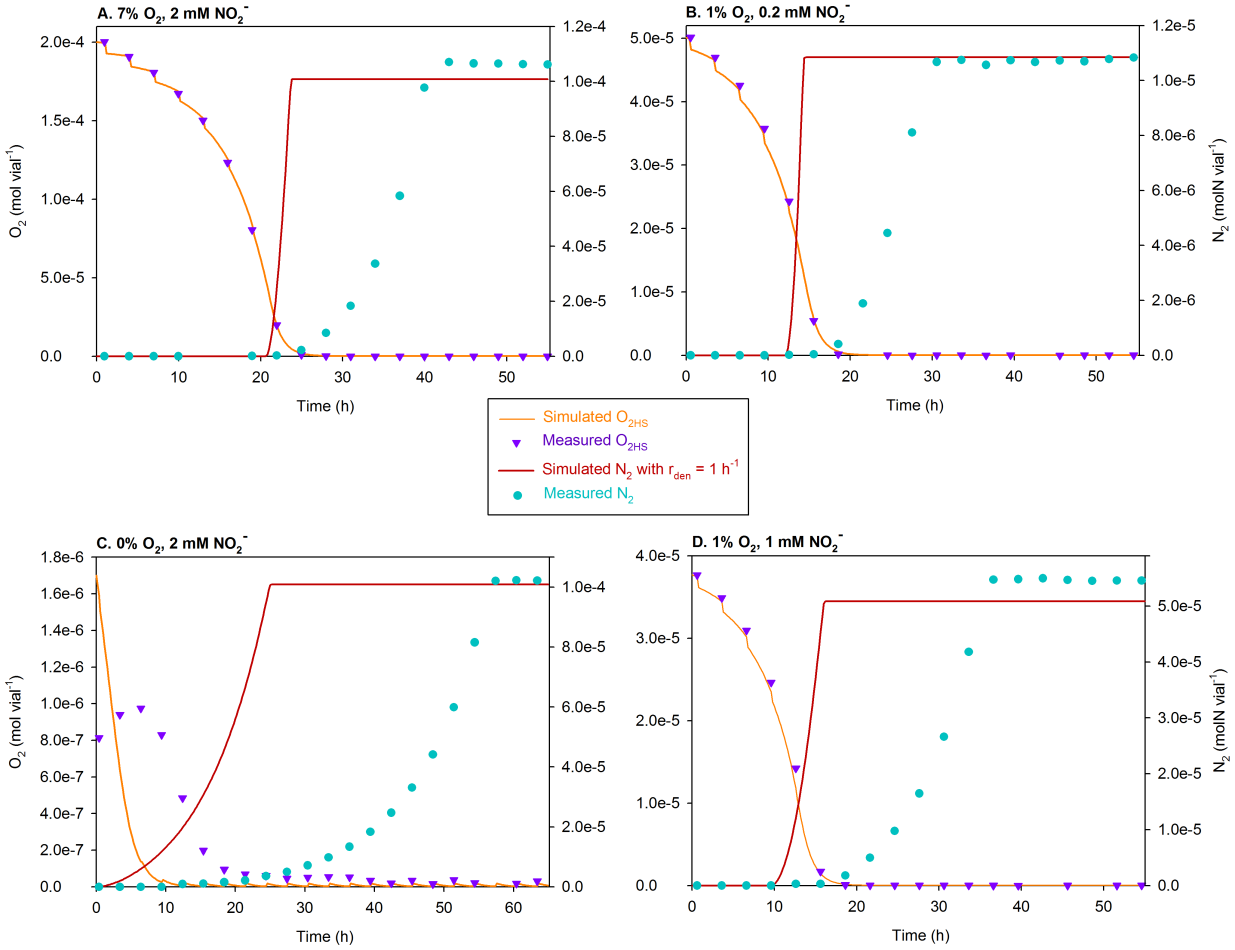
Comparison of the measured [4], [8] and simulated data assuming 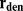 = 1 h^−1^. Assuming a single homogeneous population, as we forced our model to achieve 100% recruitment to denitrification by setting the specific-probability of recruitment (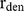) to 1 h^−1^, the simulated N_2_ accumulation (molN vial^−1^) showed considerable overestimation as compared to that measured. To illustrate this, the simulated and measured data are compared here for some randomly chosen treatments. Initial vol.% O_2_ in the headspace and initial 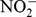 is shown above each panel.

To test the assumption of a single homogeneous population, we forced our model to achieve 100% recruitment to denitrification by setting 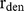 = 1 h^−1^. In consequence, the simulated N_2_ accumulation (molN vial^−1^) showed gross overestimation as compared to the measured for all the treatments (as illustrated for some randomly selected ones in Fig. 6).

**Figure 7:**
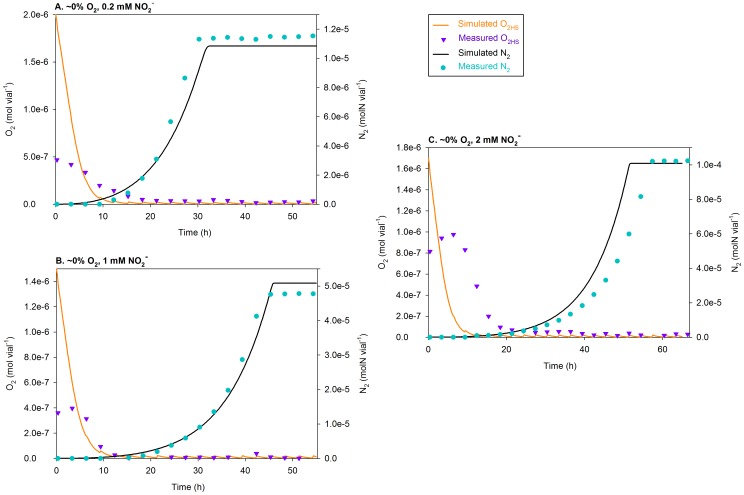
Simulations of the treatments with ∼0 vol.% 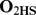 using 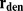 = 0.0052 h^−1^. The figure compares the measured and simulated O_2_ depletion (mol vial^−1^) and N_2_ accumulation (molN vial^−1^) for the ∼0 vol.% O_2_ treatments of Bergaust *et al.* [4], [8], i.e., the vials with near-zero O_2_ in the headspace (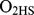) at the time of inoculation. Separate plots are shown for each initial concentration of 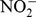 (0.2, 1, and 2 mM). The measured initial O_2_ was somewhat erratic due to episodes of needle clogging and/or high O_2_ leakage during sampling, so the initial 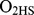 used in the simulations is chosen somewhat *ad lib* so that the simulated O_2_ depletion coincides with that measured. The discrepancy compared to the measured O_2_ seems to be significant for 2 mM 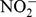 treatment. That is most likely due to the inhibitory effect of nitrite on aerobic respiration, which is not taken into account; all simulations are run with an identical 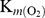. Near exhaustion, the simulated O_2_ increases slightly at each sampling time; that is due to the leakage of O_2_ via the injection system exceeding dilution of the headspace (with He) during each sampling.

**Figure 8:**
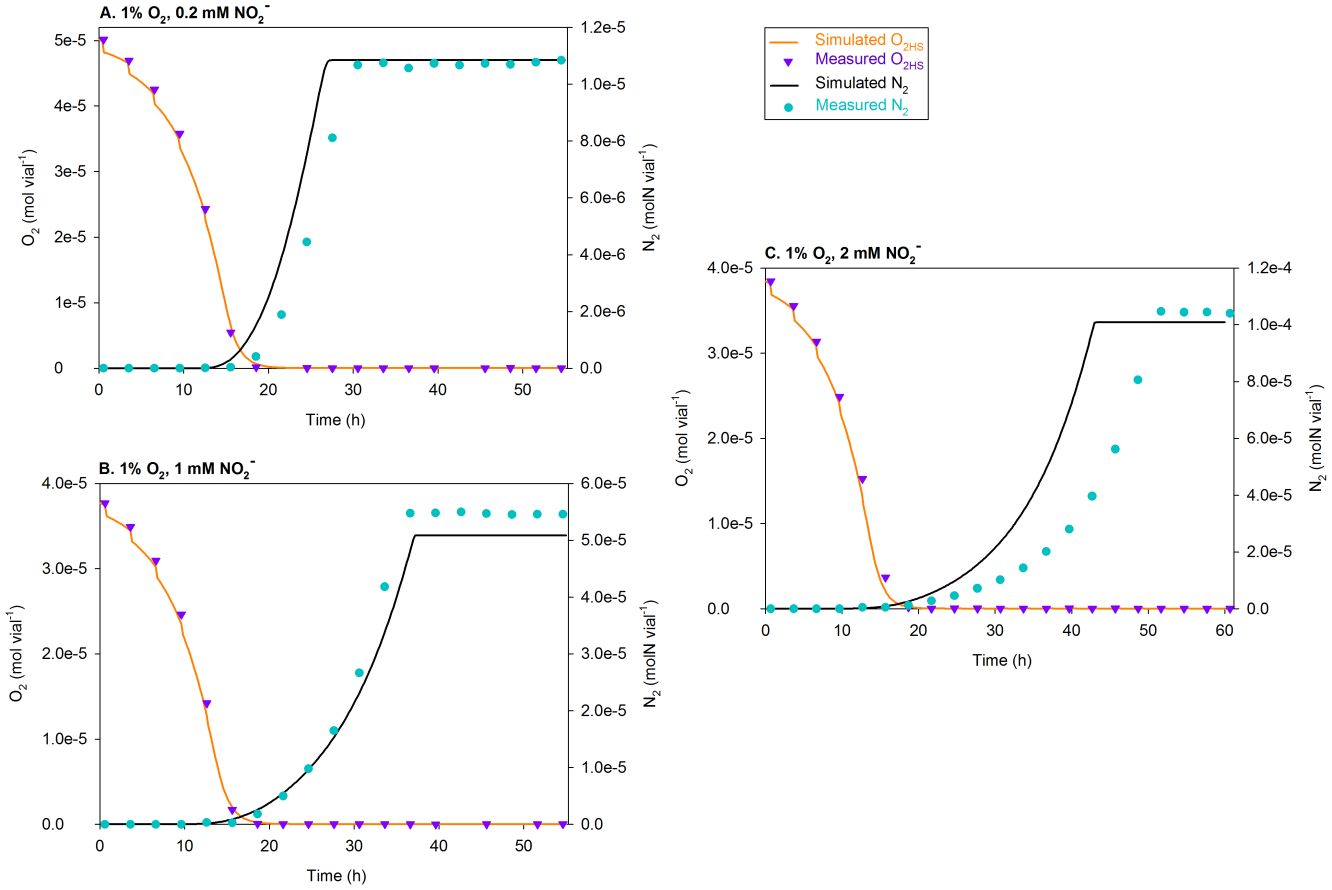
Simulations of the treatments with 1 vol.% 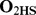 using 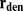 = 0.0052 h^−1^. The figure compares the measured and simulated O_2_ depletion (mol vial^−1^) and N_2_ accumulation (molN vial^−1^) for the treatments with 1 vol.% O_2_ in the headspace (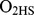) at the time of inoculation; separate plots are shown for each initial concentration of 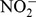 (0.2, 1, and 2 mM). At each sampling time, the simulated O_2_ is visibly reduced; that is because sampling implies 3.4% dilution of the headspace (with He). This contrasts with the simulations of the treatments with low O_2_ (Fig. 7), where the leakage of O_2_ into the system is more dominant.

**Figure 9:**
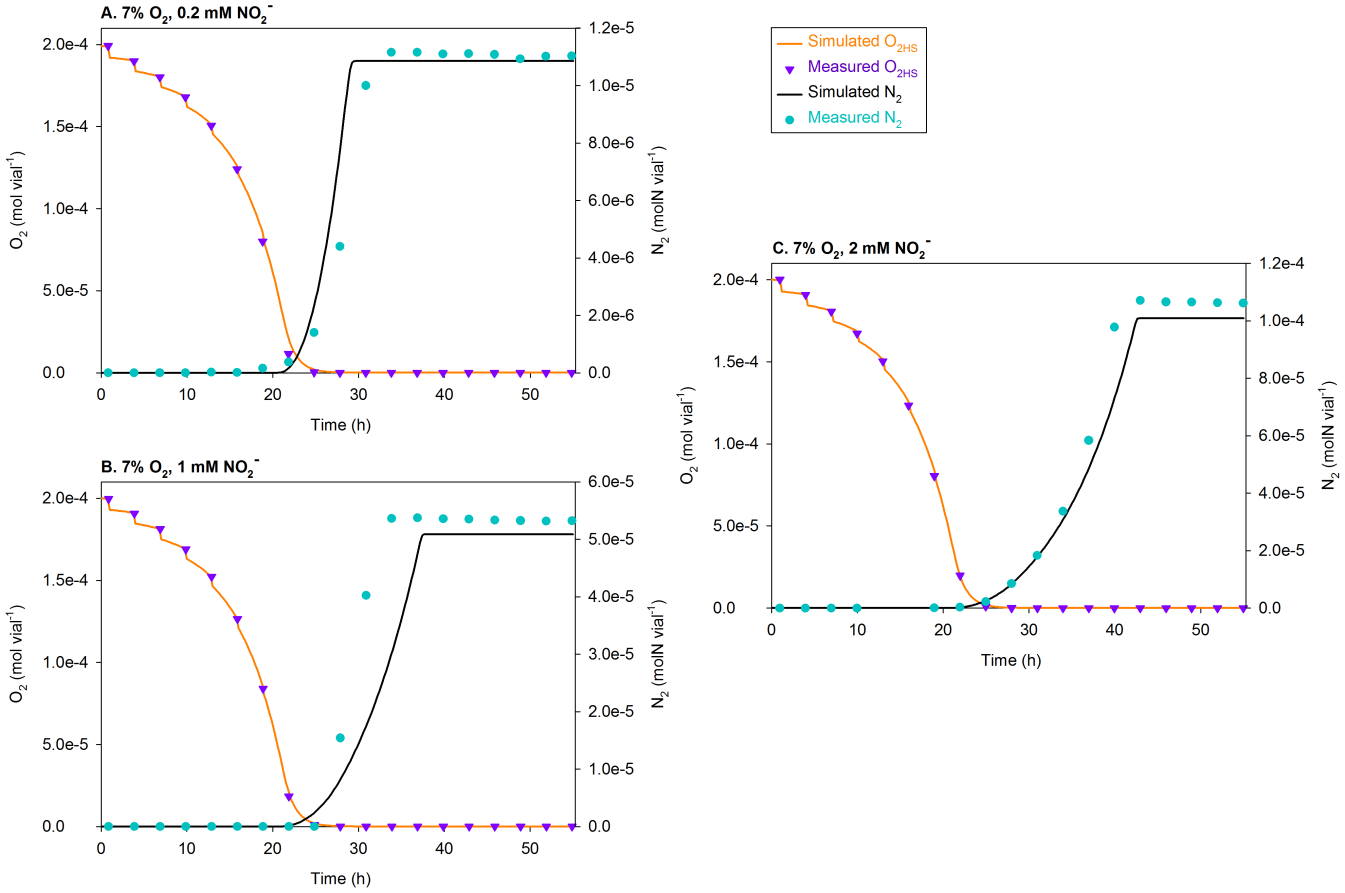
Simulations of the treatments with 7 vol.% 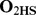 using 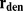 = 0.0052 h^−1^. The figure compares the measured and simulated O_2_ depletion (mol vial^−1^) and N_2_ production (molN vial^−1^) for the treatments with 7 vol.% O_2_ in the headspace (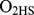) at the time of inoculation; separate plots are shown for each initial concentration of nitrite (0.2, 1, and 2 mM). At each sampling time, the simulated O_2_ is visibly reduced because of sampling, which results in 3.4% dilution of the headspace (with He).

To find a more adequate value, 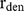 was calibrated to produce the best possible match between the simulated and measured N_2_ through optimisation. (The optimisation was carried out in Vensim DSS 6.2 Double Precision, http://vensim.com/). Table 4 presents the optimal 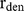 for each treatment; no consistent effect of initial [O_2_] and [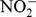] was found on the optimal results. The average for all the treatments = 0.0052, which appears to give reasonable fit between the simulated and measured N_2_ (See Figs. 7, 8, and 9). This indicates that the simulations with 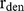 = 0.0052 should provide a reasonable approximation of 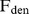 (the fraction recruited to denitrification) during the actual experiment.

**Table 4:** Specific-probability of recruitment of a cell to denitrification (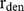) estimated for each batch culture by optimisation (best match between the simulated and measured N_2_ kinetics).

Batch No.	Treatment*: 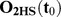 (vol.%) 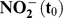 (mM)	Optimal 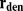 (h^−1^)
1	∼0, 0.2	0.0066
2	∼0, 1	0.0059
3	∼0, 2	0.0029
4	1, 0.2	0.0033
5	1, 1	0.0062
6	1, 2	0.0020
7	7, 0.2	0.0018
8	7, 1	0.0117
9	7, 2	0.0066
		**Avg. = 0.0052**

*Treatment refers to the initial concentration of O_2_ in the headspace (measured as headspace vol.%) and the initial concentration of 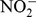 in the medium (mM).

#### Sensitivity analysis


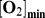
 (the O_2_ concentration below which the recruitment is arrested) was arbitrarily chosen to be 1×10^−9^ mol L^−1^. In order to evaluate the sensitivity of the model to this parameter, we tested the model performance by increasing and decreasing 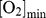 by one order of magnitude. For each parameter value, we estimated 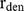 for the individual vials by optimisation (as outlined in the foregoing paragraph). A good fit was obtained for both the 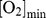 values, but the optimisation resulted in slightly different 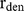 values. Increasing 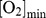 by a factor of 10 (to 1×10^−8^ mol L^−1^) resulted in 18–38% higher 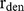 estimates (average = 28% ±stdev 10). Decreasing 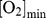 by a factor of 0.1 (to 1×10^−10^ mol L^−1^) resulted in 5–17% lower 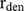 estimates (average = 11% ±stdev 6).

### The fraction recruited to denitrification (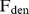)

#### A refined estimation with the presented model

Bergaust *et al.* [8], [16] and Nadeem *et al.* [9] used data from batch cultivations of *Pa. denitrificans*, as illustrated in Fig. 1, to assess 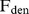. Their estimation was effectively 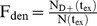, where 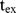 is the time when O_2_ is exhausted, 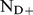 (cells vial^−1^) is the number of actively denitrifying cells estimated by the measured rate of denitrification (molN h^−1^) divided by the cell-specific denitrification (molN cell^−1^ h^−1^), and N is the total number of cells estimated on the basis of O_2_ consumption. Although this equation indisputably estimates the fraction of the cells that was actively denitrifying at the time 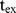, it is a biased estimate of the ‘true’ 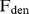 because the number of cells does not remain constant through the recruitment phase: 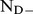 (the cells without denitrification enzymes) and 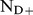 will both grow until O_2_ is depleted, but 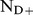 will grow faster because their growth is supported by both O_2_ and NO_x_. As a result, the estimation of 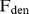 by this equation might be too high. Besides, the experimental estimation is prone to error because of infrequent sampling, since the sampling time does not necessarily coincide with 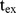.

In contrast, our model directly and more precisely calculates 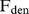 (Eq. 9) by ***a)*** explicitly simulating the actual kinetics of the recruitment of the cells to denitrification (in contrast to estimating total and denitrifying cell numbers from gas kinetics) and ***b)*** avoiding aerobic and anaerobic growth of the cells. Table 5 shows the model's estimations of 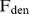 and the time-span of the recruitment (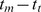) along with the 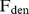 estimations of Bergaust *et al* [8], [16].

**Table 5:** The model's and Bergaust *et al.'s* [16] estimations of the fraction recruited to denitrification (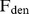).

Batch No.	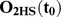 (vol.%) 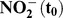 (mM)	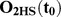 (µmol)*	Model-based Estimations		Estimations of [16]
			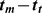 **	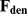	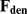
1	0, 0.2	2	25.8	0.141	0.19
2	0, 1	1.5	29.2	0.161	0.21
3	0, 2	1.7	27.2	0.156	0.19
4	1, 0.2	50.1	10.1	0.052	0.03
5	1, 1	37.8	11.1	0.056	0.07
6	1, 2	38.4	11.3	0.057	0.04
7	7, 0.2	199	7.4	0.038	0.02
8	7, 1	200	7.4	0.038	0.07
9	7, 2	200	7.4	0.038	0.08
				**Avg. = 0.082**	**Avg. = 0.1**

*Refers to the initial values of O_2_ in the headspace (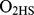) used in the simulations. The values show some inconsistency for the treatments corresponding to the same vol.% because of traces of O_2_ left behind after He-washing.

**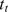 is the time when [O_2_] in the liquid falls below 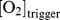 ( = 9.75 µM [22], the concentration below which recruitment of the cells to denitrification is assumed to trigger), and 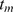 is the time when [O_2_] in the liquid falls below 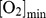 ( = 1 nM, a practically zero concentration below which the recruitment is assumed to terminate). Due to low cell density in the ∼0% O_2_ treatments, the O_2_ leakage into the vial during sampling (every 3 hours) caused oxygen concentration to exceed 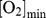 for 0.1–2.4 hours. This resulted in various recruitment spikes after the initial O_2_ was depleted. If such recruitment is omitted, 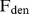 = 0.126, 0.142, and 0.133 for the treatments 1, 2, and 3, respectively.

#### In the ∼0% O_2_ treatments, 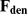 is supported by the sampling leaks of O_2_

Due to low cell density in the ∼0% O_2_ treatments (initial O_2_ = 1.5–2 µmol), the O_2_ leakage into the vial during sampling (every 3 hours) caused oxygen concentrations to exceed 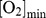 for 0.1–2.4 hours. This resulted in various spikes of recruitment after the initial O_2_ was depleted. The recruitment through these spikes amounted to, on average, ∼19% of 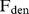 in the ∼0% O_2_ treatments.

#### 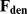
<<100%

The model's estimations of 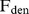 (Table 5) corroborate the suggestion of Bergaust *et al.* [8], [16] and Nadeem *et al.* [9] that in batch cultures of *Pa. denitrificans* 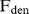 remains far below 100%. According to Bergaust *et al.* [8], [16], 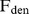 was 2–21% (average = 10%), whereas the model estimated it between 3.8–16.1% (average = 8.2%).

#### 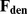 is inversely related to cell density

Bergaust *et al.* [16] argued that as the velocity of O_2_ depletion is proportional to cell density, the time-frame available for the cells to produce (necessary initial) denitrification proteome would be inversely related to the cell density at the time of O_2_ depletion. Simulation results (Table 5) support this: high initial O_2_ concentrations resulted in high cell densities at the time of O_2_ depletion, shortening the time-span for the recruitment to denitrification, hence resulting in the low 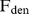.

#### Underlying cause of the low 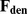



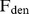
 remains low because of ***a)*** the limited time-window available to the cells for the recruitment and ***b)*** the low 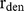 (specific-probability of the recruitment), presumably due to a low probability of initiating *nirS* transcription (subsequently reinforced through positive feedback by NO).

### Simulation of the ‘diauxic lag’

To investigate whether the recruitment of a small fraction of the cells to denitrification could explain the ‘diauxic lag’ observed by Liu *et al.* [24], we used our model to simulate the conditions they reported for their experiment. In short, Liu *et al.* [24] incubated *Ps. denitrificans* (ATCC 13867) in oxic batch cultures, which were sparged with N_2_ as the cultures had reached different cell densities (OD_550_ = 0.05–0.17). The sparging resulted in apparent diauxic lags, i.e., periods with little or no detectable growth. The length of such lags increased with the cell density present at the time of sparging.

#### Structural amendments and parameterisation of the model

To tentatively simulate their experiment, two changes were made in the O_2_ kinetics sector (Fig. 4A). Firstly, the net sampling loss of 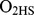 (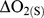) was omitted, since it was specifically set up for the robotised incubation system [28] used by Bergaust *et al* [4], [8]. Secondly, a sparging event was introduced, which immediately takes 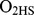 down to very low levels ( = 1×10^−9^ mol vial^−1^). Since we lack information about the exact concentration of O_2_ immediately after the sparging, the present exercise is only qualitative.

Liu *et al.* [24] inoculated the culture to have an initial OD_550_ = 0.07, which would correspond to ∼6.5×10^9^ cells vial^−1^ [4], [8]. We used this number to initialise the 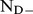 pool (shown in Fig. 4B). They used 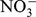 ( = 157 µmolN vial^−1^) instead of 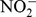, so we replaced the 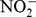 pool (Fig. 4C) by the 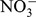 pool, initialised it accordingly, and adjusted Eqs. 11 and 15: In Eq. 11, 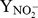 was replaced with the cell yield per molN of 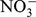 as the e^−^-acceptor (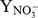 = 9.65×10^13^ cells molN^−1^ [4], [8]). In Eq. 15, 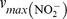 was replaced with the maximum cell-specific velocity of 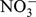 consumption (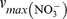 = 2×10^−15^ molN cell^−1^ h^−1^), calculated using the maximum specific NO_x_-based growth rate ( = 0.322 h^−1^) reported for their experiment. Finally, in Eq. 4, 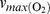 was calibrated ( = 2.28×10^−15^ mol cell^−1^ h^−1^) with the reported maximum specific aerobic growth rate ( = 0.342 h^−1^).

#### The ‘diauxic lag’ is plausibly the initial growth phase of a minute 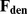 (fraction recruited to denitrification)

**Figure 10:**
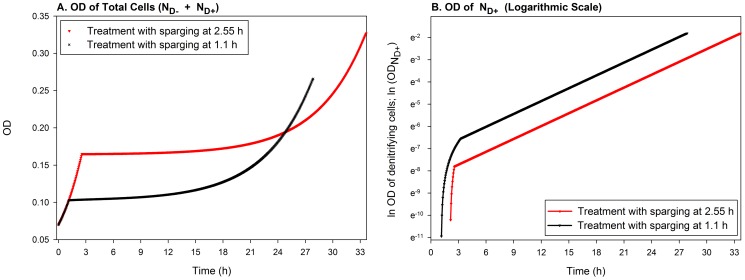
Simulation of the ‘diauxic lags’ observed by Liu *et al* [24]. A. The panel shows cumulated OD (optical density) of the cells without (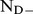) and with (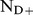) denitrification enzymes for the simulated experiment of Liu *et al.* [24], where one treatment was sparged at time = 2.55 h and the other at 1.1 h. The simulations show, qualitatively, similar ‘lags’ in the two ODs as observed by the experimenters. These apparent lags are due to exponential growth of a minute fraction of the cells that successfully switched to denitrification. The growth of this fraction remains practically undetectable (the “lag” phase) until it reaches a level comparable to the large population trapped in anoxia. B. This panel isolates the ODs of 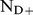 and show them on a logarithmic scale so that the exponential growth of 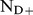, right from the onset of anoxic conditions, becomes visible. The graph initially shows a quick recruitment of the cells from the 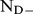 to the 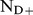 pool, followed by the exponential growth-phase.

As the experiment of Liu *et al.* [24] was simulated with the model's estimated 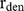 = 0.0052 h^−1^ (specific-probability of recruitment), 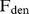 turned out to be 1.1% for the treatment sparged at h = 1.1 and 0.2% for the one sparged at h = 2.55. Simulations of the total cell density (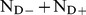) for these cases(Fig. 10A) showed long apparent lags comparable to 10–30 h lag phases observed in their later experiments [25]. However, lags in the range that Liu *et al.* [24] observed ( = 3 and 6 h for sparging at h = 1.1 and 2.55, respectively) could be achieved by our model by assuming higher residual O_2_ concentrations after sparging (resulting in a higher 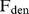). Fig. 10B isolates the OD of 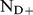 for the simulated treatments and shows them on a logarithmic scale so that their exponential growth, right from the onset of anoxic conditions, becomes apparent. The figure initially shows a quick recruitment of the cells from the 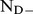 to the 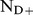 pool, followed by the exponential growth-phase of 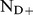.

This exercise serves to illustrate that the ‘diauxic lags’ observed [24]–[26] may simply be a result of low recruitment to denitrification in response to sudden removal of O_2_. This is possibly a more plausible explanation than suggested by the authors and further elaborated by Hamilton *et al.* [35], claiming that there is a true lag caused by extremely slow production of denitrification enzymes due to energy limitation. Our explanation of the apparent diauxic lag is corroborated by a chemostat culturing experiment conducted by Bauman *et al* [36]: A steady state carbon (acetate) limited continuous culture with *Pa. denitrificans* was made anoxic and monitored for denitrification gene transcription, N-gas production, and acetate concentrations. A transient (8–10 h) peak of acetate accumulation after O_2_ depletion suggested an apparent diauxic lag in the metabolic activity, but denitrification started immediately and increased gradually throughout the entire ‘lag’ period. They further observed that the number of denitrification gene transcripts peaked sharply during the first 1–2 hours. These observations are in good agreement with our model.

The aforestated observation of Liu *et al.* [24] that the length of the apparent lags increased with the aeration period (or the cell density at the time of sparging) is also in agreement with our model demonstrating that the time available for the cells to switch to denitrification is inversely related to the cell density at the time of O_2_ depletion.

### Model-based hypothesis: Initial O_2_ determines the timespan to denitrify all 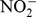 to N_2_ in a batch

Two sensitivity analyses were run to investigate the system's response to initial O_2_ in the headspace, 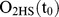: one corresponding to a range of initial [O_2_] in the liquid-phase 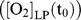 below 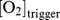 (see Eqs. 7–8) and the other for a range much higher than 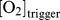. All other model parameters and initial values remained as listed in Tables 2 and 3, respectively. The exercise helps illustrate the relative importance of aerobic growth versus the recruitment (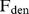) in determining the time taken to deplete the 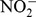 pool.

#### Sensitivity analysis (1)

Sensitivity analysis (1) was run with three 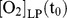 within a very low range, starting from a concentration marginally below 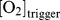:

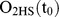
 = 2.02×10^−5^ mol vial^−1^ 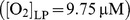,

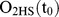
 = 1.01×10^−5^ mol vial^−1^ 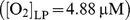,

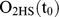
 = 5.04×10^−6^ mol vial^−1^ 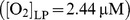

**Figure 11:**
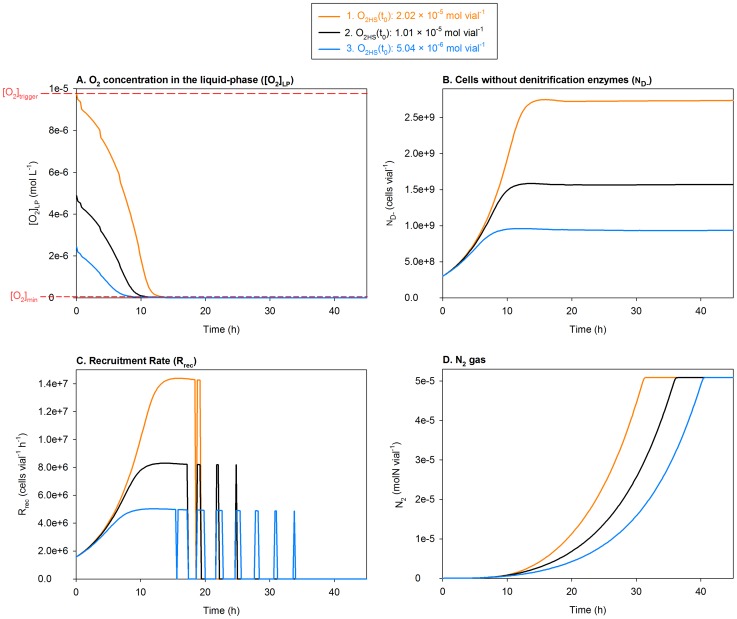
Sensitivity analysis (1): Varying initial O_2_ in the headspace 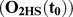 within a low range. The figure shows the impact of varying 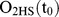 within a low range on: **A.** O_2_ concentration in the liquid-phase 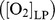, **B.** The number of aerobically growing cells (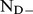), which do not possess denitrification enzymes, **C.** The rate of recruitment of 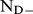 to denitrification (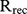), and **D.** N_2_ accumulation. Marked in Panel A, 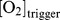 is the 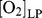 below which 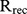 triggers, and 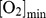 is the 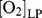 below which 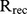 terminates. In Panel C, the spikes of recruitment (following the initial recruitment) are due to spikes of O_2_ by sampling, causing 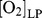 to transiently exceed 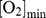. The model predicts that reducing 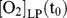 within a low range (Panel A) will lower the number of aerobically grown cells (Panel B) and, thereby, the recruitment rate (Panel C), thus increasing the time taken to deplete 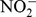 (slower N_2_ accumulation, Panel D).

This is rather a simple case demonstrating that increasing 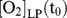 within this low range (Fig. 11A) will result in increasing rates of denitrification (Fig. 11D) by increasing the number of aerobically grown cells (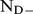, Fig. 11B) and, thus, the rate of recruitment (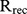, Fig. 11C).

#### Sensitivity analysis (2)

Sensitivity analysis (2) was run with three initial O_2_ concentrations much higher than 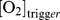:

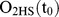
 = 2×10^−4^ mol vial^−1^ 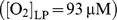,

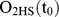
 = 1.19×10^−4^ mol vial^−1^ 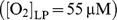,

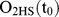
 = 3.84×10^−5^ mol vial^−1^ 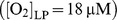

**Figure 12:**
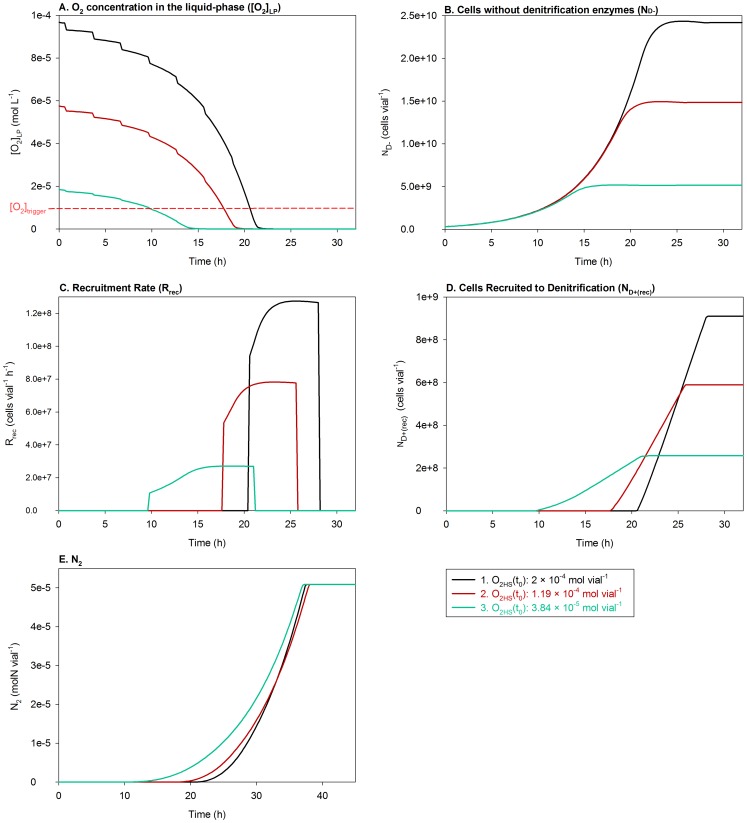
Sensitivity analysis (2): Varying initial O_2_ in the headspace (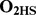
(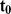)) within a high range. The figure shows the impact of varying 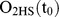 within a range much higher than 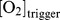 (the [O_2_] below which recruitment of the cells to denitrification is assumed to trigger) on: **A.** O_2_ concentration in the liquid-phase 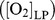, **B.** The number of aerobically growing cells (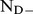), which do not possess denitrification enzymes, **C.** The rate of recruitment of 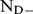 to denitrification (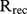), **D.** The number of cells as a result of the recruitment alone (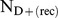), i.e., the denitrifying cells (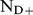) but without aerobic and NO_x_-based growth, and **E.** Cumulated N_2_. The cumulated N_2_ reached stable plateaus at nearly the same time for all the runs (Panel E), despite the fact that the time taken to deplete O_2_ below 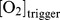 decreased with increasing 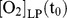 (Panel A). Thus, once denitrification was initiated, the rates increased with increasing initial 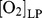 due to an increasing population of oxygen-grown cells (Panels B–D). The fraction of the cells recruited to denitrification (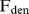) declined with increasing initial O_2_ concentration (not shown), but this was not sufficient to compensate for the increasing number of oxygen-raised cells.

In this case, the cumulated N_2_ reached stable plateaus at nearly the same time for all the runs (Fig. 12E), despite that the time taken to deplete O_2_ below 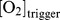 decreased with increasing 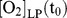 (Fig. 12A), reducing the time available to the cells for switching to denitrification (See Fig. 5). Thus, once denitrification was initiated, the rates increased with increasing 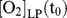 due to an increasing population of oxygen-grown cells (Fig. 12B–D). 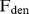 (Eq. 9) declined with increasing 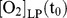 (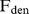 = 0.058, 0.041 and 0.028 for runs 3, 2 and 1, respectively), but this was not sufficient to compensate for the increasing number of oxygen-raised cells.

If the model is run without any initial O_2_, there would be no recruitment and, hence, no denitrification. Verification of this in batch cultures is difficult because traces of O_2_ remain after He-washing of the batches. However, we (Bergaust *et al.*, unpublished data) have been able to demonstrate that the aerobically grown *Pa. denitrificans* cells are indeed entrapped in anoxia if transferred to anoxic conditions as instantaneously as in the experiments conducted by Højberg *et al.* [15].

### Conclusion

The prevailing wisdom in denitrification research is that, under impending anoxic conditions, *all* cells in a batch culture of denitrifying bacteria will switch to denitrification. However, recent experiments with batch cultures of *Pa. denitrificans* have provided evidence that, in response to O_2_ depletion, only a small fraction (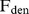) of the entire population is able to switch to denitrification [4], [8], [9]. The evidence is based on indirect analyses of e^−^-flow rates to O_2_ and NO_x_ during the transition of the cells from aerobic to anaerobic respiration. To provide a direct and refined estimation of 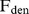, we constructed a dynamic model and directly simulated kinetics of recruitment of the cells to denitrification. We first formulated a hypothesis as to the underlying regulatory mechanism of cell differentiation under approaching anoxia. Briefly, it is that the low 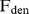 is due to a low probability of initiating transcription of the *nirS* genes, but once initiated, the transcription is greatly enhanced through autocatalytic positive feedback by NO, resulting in the recruitment of the transcribing cell to denitrification. Then, as we implemented this hypothesis in the model, the simulation results showed that the specific-probability (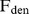) of 0.0052 (h^−1^) for a cell to switch to denitrification is sufficient to robustly simulate the measured denitrification gas kinetics. The model estimated the resultant 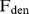 between 3.8–16.1% only (average = 8.2%). The phenomenon may be considered as a ‘bet-hedging’ regulation ‘strategy’ [12]: the fraction switching to denitrification benefits if the anoxic spell is long and NO_x_ remains available, whereas the non-switching fraction benefits, by saving energy required for the protein synthesis, if the anoxic spell is short. The strategy has important implications for the interpretation of numerous experiments on *Pa. denitrificans* and other denitrifying organisms, as this study has illustrated by presenting a more plausible explanation of the apparent diauxic lags [24] on the basis of the low 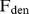.

## Supporting Information

Dataset S1 contains a Vensim simulation model (Hassan_et_al_2014.mdl) used in this study along with two files (7%_Oxygen_2mM_Nitrite.vdf and Measured_Data) containing simulated and measured data, respectively.
(ZIP)
